# Impact of hemo-alert program on appropriate antimicrobial therapy in hospitalized patients with bacteremia: a quasi-experimental study

**DOI:** 10.1017/ash.2026.10757

**Published:** 2026-07-29

**Authors:** Siwakan Phuetphaichit, Surangkana Samanloh, Witchuda Kamolvit, Pinyo Rattanaumpawan

**Affiliations:** 1 Department of Medicine, Faculty of Medicine Siriraj Hospital, Mahidol University, Bangkok, Thailand; 2 Clinical Trial Unit, Research Department, Faculty of Medicine Siriraj Hospital, Mahidol University, Bangkok, Thailand; 3 Department of Microbiology, Faculty of Medicine Siriraj Hospital, Mahidol University, Bangkok, Thailand; 4 Division of Infectious Diseases and Tropical Medicine, Department of Medicine, Faculty of Medicine Siriraj Hospital, Mahidol University, Bangkok, Thailand

## Abstract

**Objectives:**

To evaluate the impact of the HemoAlert program—a notification system providing pathogen identification and antimicrobial susceptibility results—on the time to initiation of appropriate antimicrobial therapy in patients with bacteremia.

**Design:**

Pre–post-quasi-experimental study.

**Setting:**

Siriraj Hospital, the largest tertiary care hospital in Bangkok, Thailand.

**Patients:**

Adult hospitalized patients with at least one positive blood culture between December 2021–February 2022.

**Methods:**

A quasi-experimental study was conducted to assess the impact of the HemoAlert program, which delivered SMS notifications of final microbiology results to the responsible physician and updated the patient’s medical record. The primary outcome was the time to appropriate antimicrobial therapy. Secondary outcomes included appropriateness of antimicrobial therapy at 72 and 120 hours, favorable clinical outcome, 28-day mortality, and length of hospital stay.

**Results:**

A total of 150 patients were included in both periods. Demographics and pathogen distribution were similar between groups. The most common pathogens were *Escherichia coli, followed by Klebsiella pneumoniae*, and *Staphylococcus aureus.* The postimplementation group also showed a trend toward a shorter time to appropriate antimicrobial therapy (81.38 ± 61.38 vs 66.17 ± 51.81 h; *P* = .08). Patients in the postimplementation group were more likely to receive appropriate therapy within the first 72 hours (34.0% vs 22.7%; *P* = .03). Favorable clinical response was more frequent after implementation (74.0% vs 85.3%; *P = .02*), whereas 28-day mortality was similar (24.0% vs 18.0%; *P = .20*).

**Conclusion:**

The HemoAlert program improved the timeliness and appropriateness of antimicrobial therapy and enhanced clinical response in patients with bacteremia, supporting the utility of simple notification systems in optimizing management.

## Introduction

Bacteremia is a major global public health concern. Each year, nearly two million cases occur in North America and Europe, resulting in approximately 250,000 deaths.^1^ Without timely or appropriate management, bacteremia can progress to sepsis—a life-threatening condition in which the body mounts a dysregulated response to infection, leading to organ dysfunction, severe morbidity, and death. Globally, sepsis remains one of the leading causes of mortality, accounting for an estimated 20% of all deaths.^2^

Previous studies have shown that delayed initiation of appropriate antimicrobial therapy in patients with sepsis—particularly those with bacteremia—is significantly associated with poor clinical outcomes, including prolonged hospitalization and increased morbidity and mortality.^3–7^ It is recommended that serious infections, including bacteremia, be treated empirically with broad-spectrum antimicrobial therapy, followed by de-escalation to a narrower-spectrum regimen once the causative pathogen and its susceptibility profile are identified.^8^ For patients with bacteremia who demonstrate clinical improvement, targeted therapy may be considered to reduce unnecessary exposure to broad-spectrum antibiotics.^8^

There is no standard definition of appropriate antimicrobial therapy. According to the general principles of antimicrobial use, the selection of therapy should take into account multiple factors, including the causative pathogen, the type and route of the antimicrobial agent, the site of infection, and the patient’s underlying or specific clinical conditions.^9^ These considerations aim to maximize treatment response, minimize adverse effects, and preventing the emergence of antimicrobial resistance.

Based on an observational study conducted at our hospital, appropriate antibiotics were administered within 6 hours in only 39% of patients with sepsis, of whom approximately one-third also had bacteremia.^10^ Delays in initiating appropriate antimicrobial therapy may result from various factors, including limited knowledge among prescribing clinicians, restricted access to effective antimicrobial agents, limitations in laboratory diagnostics and reporting, diagnostic uncertainty, high patient load, workflow inefficiencies, communication gaps, and patient-specific complexities.^11–13^

Several studies have demonstrated the benefits of hemoculture notification systems in patients with bacteremia, including improved antibiotic appropriateness, reduced time to appropriate therapy, decreased mortality, shorter hospital length of stay, and lower readmission rates.^14–17^ They utilized either paper-based or automatic computerized alert systems, occasionally paired with phone recommendations from an infectious disease specialist.^17^ These systems operated alongside either rapid or standard identification methods.^14^ Evidence remains limited regarding manual alert systems and their application in resource-limited settings.

Currently, Siriraj Microbiology Laboratory reports blood cultures in two steps: first, immediate phone notification of positive Gram stain results to the ward nurse; second, logging final biochemical identification and susceptibility results into the electronic database, where they must be manually retrieved by the responsible physician. Because there is no secondary notification for the final results, the antimicrobial adjustment may be delayed. Given these limitations, implementing an intervention to improve the appropriateness of antimicrobial therapy is necessary. Therefore, we conducted a quasi-experimental study to evaluate the impact of the Hemo-Alert program on time to appropriate antibiotic therapy and clinical outcomes among hospitalized adult patients with bacteremia at Siriraj Hospital.

## Methods

### Study design and setting

This quasi-experimental, pre–post-implementation study was conducted among all hospitalized adults (age ≥18 yr) with at least one positive blood culture with any organisms at Siriraj Hospital between December 2021–February 2022. Following the launch of the Hemo-Alert program on January 1, 2022, and a subsequent two-week washout period, participants were divided into two cohorts: the preimplementation group (1–31 December 2021) and the postimplementation group (17 January–28 February 2022).

Siriraj Hospital is a 2,300-bed university hospital located in Bangkok, Thailand. The study protocol was approved with a waiver of informed consent by the Institutional Review Board of the Faculty of Medicine, Siriraj Hospital, Mahidol University (Certificate of Approval No. Si 885/2021) in November 2021. The study was part of a quality improvement program at our hospital.

### Microbiology standard practices and the hemo-alert program

Before the implementation of the Hemo-Alert program, standard microbiology practice involved notifying the ward nurse of a positive blood culture result via phone call. However, the final pathogen identification and antimicrobial susceptibility results had to be manually retrieved from the electronic laboratory system by the responsible physician.

The workflow of the Hemo-Alert program was as follows: after obtaining a blood culture specimen, the hospital number and laboratory number were entered into the microbiology laboratory database. Once the automated blood culture system signaled a positive result, pathogen identification and antimicrobial susceptibility testing were performed. The microbiology staff then sent the report via an automated email system to the Hemo-Alert team, who subsequently delivered the final results via Short Message Service (SMS) to the responsible staff and ensured the report was placed on the first page of the patient’s medical paper chart (as shown in Supplementary File 1). The Hemo-Alert team consisted of ID fellows, an AMS pharmacist, and ID staff physicians.

### Data collection

Patient clinical data were obtained via paper chart review, and laboratory data were retrieved from the electronic laboratory system. The following information was collected: baseline characteristics (age, weight, sex, admitting ward, prior hospitalization within the past three months, underlying diseases, and source of infection), clinical parameters, laboratory data, microbiological data, and antimicrobial treatment data.

### Outcome assessment and definitions

Appropriateness of antimicrobial therapy was independently assessed by two blinded internal medicine specialists through review of patient paper charts and microbiological data. Appropriate antimicrobial therapy was defined as administration of the first dose of an antimicrobial agent with activity against the identified pathogen, adequate penetration to the site of infection, and no unnecessarily broad-spectrum coverage. Discontinuation of antibiotics in cases where blood cultures grew contaminants was also considered appropriate antimicrobial therapy. Discrepancies were resolved by a third reviewer, an infectious disease physician. Inappropriate regimens were categorized according to specific reasons, including inappropriate spectrum, incorrect dosing, or excessive duration. All reviewers remained blinded to group allocation.

**Appropriateness of antimicrobial therapy** was evaluated at 72 and 120 hours after blood culture collection using blood culture results alone and in combination with microbiological results from other infection sites.

**Time to appropriate antimicrobial therapy** was the interval from blood culture collection to the first dose of an appropriate agent.

**Favorable clinical response** was defined as the resolution of all clinical symptoms without systemic inflammatory response syndrome.

**28-day mortality** was defined as death from any cause within 28 days after the date of blood collection.

### Statistical analysis and sample size calculation

Based on a pilot study of 25 bacteremic patients at Siriraj Hospital, only 56% received appropriate antimicrobial therapy within the first 72 hours. The Hemo-Alert program was expected to improve the appropriateness of therapy by 20%. Using a two-sided alpha of .05 and a beta of .20, the estimated sample size was 136 patients per group. Assuming a 10% loss to follow-up, the total sample size was set at 150 patients per group.

Demographic and baseline characteristics of eligible patients were analyzed using descriptive statistics. Categorical variables were presented as frequencies and percentages, whereas continuous variables were summarized using means and standard deviations. Between-group comparisons were performed using the χ^2^ or Fisher’s exact test, as appropriate.

The primary outcome was the time to appropriate antimicrobial therapy. Secondary outcomes included appropriateness of antimicrobial therapy at 72 and 120 hours, favorable clinical outcome, 28-day mortality, and length of hospital stay. For outcome analysis, the χ^2^ test was applied for each categorical variable. Multivariable stepwise logistic regression was used to identify independent factors associated with a favorable clinical response, including variables with a univariate *P* < .2. A *P*value of less than .05 was considered statistically significant.

## Results

**Figure 1.**
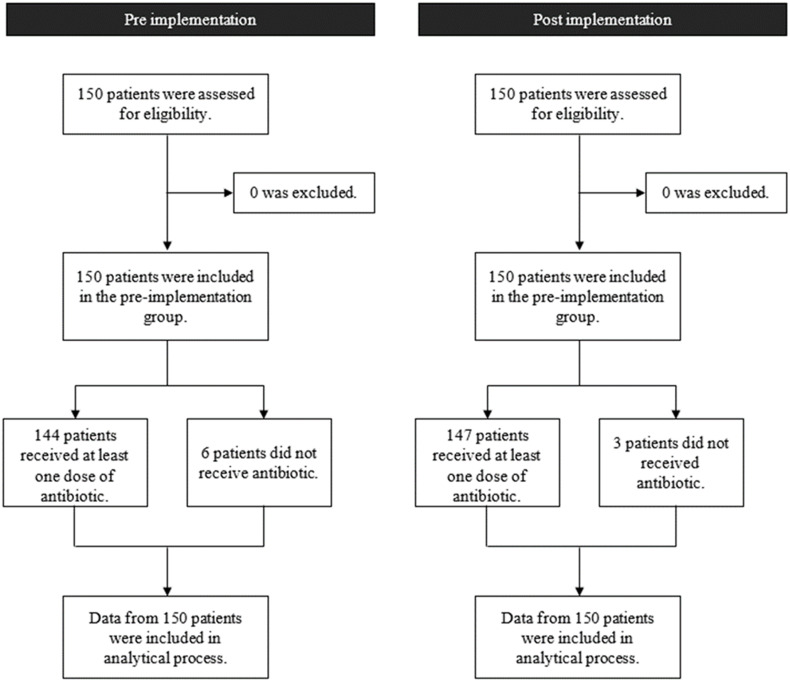
Study flow chart. Figure 1 long description.

Among 300 unique patients with positive blood cultures during the study period, 150 patients were included in each of the pre and postimplementation groups (Figure 1). Of these, 291 patients received at least one dose of antibiotics, including 144 patients in the preimplementation group and 147 patients in the postimplementation group.

### Baseline characteristics

Baseline characteristics of patients in the preimplementation and postimplementation groups are presented in Table 1. Approximately half of patients in each group were male (50.7% vs 48.7%; *P* = .73), with similar mean ages (66.1 ± 17.1 vs 68.6 ± 14.4 yr; *P* = .18). Patients in the postimplementation group were more likely to be admitted to a general medicine ward (70.0% vs 84.7%; *P* = .002) and had a higher prevalence of hospitalization within the past three months (38.0% vs 52.0%; *P* = .01). Furthermore, patients in the postimplementation group had significantly higher rates of hypertension (57.3% vs 70.7%; *P* = .02), non-malignant hematologic disease (9.3% vs 19.3%; *P* = .01), and hematologic malignancy (5.3% vs 12.0%; *P* = .04).

**Table 1.** Baseline characteristics of patients in the preimplementation group and the postimplementation group Table 1 long description.

Variables	Total (n = 300)	Preimplementation (n = 150)	Postimplementation (n = 150)	*P-value*
**Male gender**	149 (49.7%)	76 (50.7%)	73 (48.7%)	.73
**Mean age ± SD (years)**	67.36 ± 15.85	66.14 ± 17.13	68.57 ± 14.41	.18
**Previous hospitalization in the past 3 months**	135 (45.0%)	57 (38.0%)	78 (52.0%)	.01
**Admission to the general medicine ward**	232 (77.3%)	105 (70.0%)	127 (84.7%)	.002
**Having one or more comorbidities**	280 (93.3%)	135 (90.0%)	145 (96.7%)	.02
Hypertension	192 (64.0%)	86 (57.3%)	106 (70.7%)	.02
Cerebrovascular disease	68 (22.7%)	29 (19.3%)	39 (26.0%)	.17
Chronic respiratory disease	27 (12.3%)	15 (10.0%)	12 (8.0%)	.55
Cardiovascular disease	85 (28.3%)	37 (24.7%)	48 (32.0%)	.16
Diabetes mellitus	124 (41.3%)	56 (37.3%)	68 (45.3%)	.16
Chronic kidney disease	102 (34%)	48 (32%)	54 (36%)	.46
Hepatic dysfunction	44 (14.7%)	18 (12.0%)	26 (17.3%)	.19
Non-malignant hematologic disease	43 (14.3%)	14 (9.3%)	29 (19.3%)	.01
Hematologic malignancy	26 (8.7%)	8 (5.3%)	18 (12.0%)	.04
Solid malignancy	59 (19.7%)	30 (20.0%)	29 (19.3%)	.88
Prior transplantation	7 (2.3%)	2 (1.3%)	5 (3.3%)	.45*
Receipt of immunosuppressive agent	55 (18.3%)	25 (16.7%)	30 (20.0%)	.46
Neutropenia	16 (5.3%)	7 (4.7%)	9 (6.0%)	.61
HIV Infection	4 (1.3%)	3 (2.0%)	1 (.7%)	.62*
Other	22 (7.3%)	11 (7.3%)	11 (7.3%)	1.00
**Hospital-acquired infection**	138 (46%)	73 (48.67%)	65 (43.33%)	.35
Site (s) of infection**				
Bacteremia with identified source (s)	229 (76.3%)	112 (74.7%)	117 (78.0%)	.50
Catheter-related bloodstream infection	22 (7.3%)	9 (6.0%)	13 (8.7%)	.38
Urinary tract infection	74 (24.7%)	39 (26.0%)	35 (23.3%)	.59
Lower respiratory tract infection	67 (22.3%)	35 (23.3%)	32 (21.3%)	.68
Skin and soft tissue infection	27 (9.0%)	15 (10.0%)	12 (8.0%)	.55
Gastrointestinal tract infection	62 (20.7%)	27 (18.0%)	35 (23.3%)	.25
Other	12 (4.0%)	7 (4.7%)	5 (3.3%)	.56

*Note.* * *p*-values calculated using non-parametric tests; ** a patient may have multiple sites of infection.

Table 2 shows the distribution of causative pathogens of the preimplementation group versus the postimplementation group. The three most common pathogens were *Escherichia coli, Klebsiella pneumoniae*, and *Staphylococcus aureus*. Overall, pathogen distribution was similar between groups.

**Table 2.** Distribution of causative pathogens in patients during the preimplementation period and the postimplementation period Table 2 long description.

Causative pathogens	Preimplementation (n = 150)	Postimplementation (n = 150)	*P*
**Gram-positive bacteria**			
• *Enterococcus* spp.	15 (10.0%)	21 (14.0%)	.29
• *Staphylococcus* spp.	15 (10.0%)	16 (10.7%)	.85
• *Staphylococcus aureus*	14 (9.3%)	16 (10.7%)	.70
• *Methicillin-resistant strains*	0 (%)	2 (1.3%)	.16
• *Streptococcus* spp.	12 (8.0%)	9 (6.0%)	.50
• Others	1 (.7%)	1 (.7%)	1.00
**Gram-negative bacteria**			
• *Enterobacterales* spp.	60 (40.0%)	68 (45.3)	.35
• *Escherichia coli*	36 (24.0%)	40 (26.7%)	.60
• *Klebsiella pneumoniae*	21 (14.0%)	25 (16.7%)	.52
• *Extended-spectrum beta-lactamase producer*	19 (12.7%)	25 (16.7%)	.33
• *Carbapenem-resistant strain*	3 (2.0%)	8 (5.3%)	.13
• *Pseudomonas aeruginosa*	9 (6.0%)	17 (11.3%)	.10
• *Acinetobacter baumannii*	9 (6.0%)	10 (6.7%)	.81
• *Aeromonas* spp.	4 (2.7%)	2 (1.3%)	.68
• Others	25 (16.7%)	18 (12.0%)	.25
**Contaminants**	50 (33.3%)	53 (35.3)	.72
**Fungi**	0 (0%)	4 (2.7%)	.12

### Appropriateness of antimicrobial therapy and clinical outcomes

Table 3 summarizes the appropriateness of antimicrobial therapy and clinical responses in the pre and postimplementation groups. The postimplementation group showed a trend toward a shorter time to appropriate antimicrobial therapy (81.38 ± 61.38 vs 66.17 ± 51.81 h; *P* = .08). Patients in the postimplementation group were more likely to receive appropriate therapy within the first 72 hours (22.7% vs 34.0%; *P* = .03). When all infection sites were considered, appropriate therapy was also higher in the postimplementation group at 72 hours (48.7% vs 64.7%; *P* = .005) and 120 hours (48.7% vs 64.0%; *P* = .007).

**Table 3.** Outcomes of interest of patients in the preimplementation group and the postimplementation group Table 3 long description.

Outcomes	Preimplementation (n = 150)	Postimplementation (n = 150)	*P*
**Primary outcomes**			
**Mean time to appropriate antimicrobial therapy (hours) ± SD**	81.38 ± 61.38	66.17 ± 51.81	.08
**Secondary outcomes**			
Appropriate antimicrobial therapy (based on hemoculture)			
– At 72 hours	34 (22.7%)	51 (34.0%)	.03
– At 120 hours	70 (46.7%)	81 (54.0%)	.20
Appropriate antimicrobial therapy (based on hemoculture and other infection sites)			
– At 72 hours	34 (48.7%)	51 (64.7%)	.005
– At 120 hours	70 (48.7%)	81 (64.0%)	.007
Favorable clinical response	111 (74%)	128 (85.3%)	.02
28-day mortality rate	36 (24%)	27 (18%)	.20
Mean length of hospital stay **(days) ± SD**	22.43 ± 32.62	27.36 ± 35.29	.21

Favorable clinical response was significantly increased postimplementation (74.0% vs 85.3%; *P* = .02), while 28-day mortality was similar between groups (24.0% vs 18.0%; *P* = .20). The mean length of stay was comparable between the pre and postimplementation groups (22.43 ± 32.62 vs 27.36 ± 35.29; *P* = .21).

### Independent factors associated with favorable clinical response

Multivariable analysis identified four independent factors associated with a favorable clinical response. Positive predictors included postimplementation group (adjusted OR [aOR] = 8.49; 95% CI 3.56–20.23; *P* < .005), and hemoculture positive for coagulase-negative Staphylococci (aOR = 3.23; 95% CI 1.68–6.20; *P* < .005). Poor prognostic factors were carbapenem-resistant *Klebsiella pneumoniae* bacteremia (aOR = 0.09; 95% CI 0.03–0.35; *P* < .005), and admission to a general medicine ward (aOR = 0.26; 95% CI 0.10–0.66; *P* = .005).

## Discussion

Our study demonstrated that patients in the postimplementation group were significantly more likely to receive appropriate antimicrobial therapy within the first 72 hours based on hemoculture results. When considering all infection sites, the appropriateness of therapy at both 72 and 120 hours was also higher in the postimplementation group. These findings are consistent with previous studies that implemented systematic notification of positive hemoculture results to improve antibiotic appropriateness and clinical outcomes.^16,17^ Despite a higher prevalence of comorbidities, the postimplementation group showed a significantly better clinical response. Although 28-day mortality tended to be lower in this group, the difference did not reach statistical significance.

Owing to differences in baseline characteristics between groups, a multivariate analysis was performed to assess the impact of the Hemo-Alert program. The postimplementation period was identified as an independent predictor of a good clinical response. Additionally, a positive blood culture for coagulase-negative Staphylococci was also an independent predictor, likely reflecting its well-known role as a common contaminant in blood cultures.^18,19^

Carbapenem-resistant *Klebsiella pneumoniae* bacteremia was identified as an independent factor negatively associated with a good clinical response, consistent with its well-established link to high mortality in carbapenem-resistant Gram-negative infections.^20^ Additionally, admission to a general medicine ward was associated with a poorer clinical response, likely reflecting a higher severity of illness in these patients, which may not have been fully accounted for by the multivariate analysis. Similar patterns have been reported in other university teaching hospital.^21^

Based on chart review, broad-spectrum antibiotics were continued without de-escalation in 66% of postimplementation cases, despite final susceptibility results being available within 72 hours. This indicates that simply providing notifications may be insufficient to improve appropriate antimicrobial therapy if physicians lack the knowledge, understanding, or confidence to adjust treatment based on the results. Addressing this knowledge gap may be essential to achieving meaningful improvements in antimicrobial stewardship.

This study has several limitations. First, as a quasi-experimental design, the observed differences between pre and postimplementation periods may not be solely attributable to the Hemo-Alert program. To address this, a multivariate analysis was performed to adjust for potential confounders. Second, some patients had multiple infection sites, and different pathogens may have been involved. At the time hemoculture results became available, cultures from other sites may have still been pending, making it difficult to determine appropriateness. To minimize misclassification, appropriateness was assessed not only by pathogen–drug matching but also by the consensus of three independent, blinded physicians. Third, most patients were admitted to general medicine wards, limiting generalizability to other hospital settings. Lastly, our Hemo-Alert Program relies on multiple manual steps from the laboratory to the research nurse, which may limit sustainability. A fully integrated alert system directly linked to the responsible physician would likely improve efficiency and long-term feasibility.

Our study has several key strengths and clinical implications. First, we also evaluated a comprehensive suite of clinical outcomes—including favorable clinical response and mortality. Second, our analysis separated treatment appropriateness based on blood cultures alone versus combined blood and secondary infection site cultures. Finally, because our intervention operates via automated system or non-medical personnel without requiring ID physician involvement, it represents a highly scalable, resource-efficient tool for AMS in settings with limited specialist availability.

In conclusion, the Hemo-Alert program improved the appropriateness of antimicrobial therapy and clinical outcomes in patients with bacteremia by delivering timely microbiology notifications to physicians. However, the impact of such alerts also depends on physicians’ knowledge and confidence in adjusting therapy. These findings highlight the importance of combining alert systems with education to optimize antimicrobial prescribing and patient outcomes. Further studies should assess its effectiveness in other infections and clinical settings.

## Supporting information

10.1017/ash.2026.10757.sm001 Phuetphaichit et al. supplementary material Phuetphaichit et al. supplementary material

## Supplementary material

The supplementary material for this article can be found at https://doi.org/10.1017/ash.2026.10757.

## Table 1. Long description

Baseline characteristics of patients in the pre-implementation group and the post-implementation group

Navigate back to Table 1..

## Table 2. Long description

Distribution of causative pathogens in patients during the pre-implementation period and the post-implementation period

Navigate back to Table 2..

## Table 3. Long description

Outcomes of interest of patients in the pre-implementation group and the post-implementation group

Navigate back to Table 3..

## Figure 1. Long description

Study flow chart

Navigate back to Figure 1..
