# Supplementary material for: Impact of hemo-alert program on appropriate antimicrobial therapy in hospitalized patients with bacteremia: a quasi-experimental study
Source: Antimicrob Steward Healthc Epidemiol. 2026 Jul 29;6(1):e220. doi: 10.1017/ash.2026.10757 (PMC13419442; doi:10.1017/ash.2026.10757)
Supplement: Phuetphaichit et al. supplementary material [file S2732494X26107578sup001.pdf]

Short Message Service Script

Patient: [First Name] [First Letter of Last Name] in ward [Ward Name]

The identification and susceptibility test result dated [Date] is now available.

Hemo-alert Team

Example of final microbiological reports shown on the first page of patient's medical record

Blood culture

Identification: *Escherichia coli*

Susceptibility report:

Susceptibility	Isolate 1
Amikacin	R
Amoxicillin/clavulanate	R
Ampicillin	R
Cefepime	R
Cefoxitin	R
Ceftazidime	R
Ceftriaxone	R
Cefuroxime	R
Ciprofloxacin	R
Ertapenem	R
Gentamicin (10 mcg)	R
Imipenem	R
Meropenem	R
Netilmicin	R
Nitrofurantoin	R
Piperacillin/tazobactam	R
Tetracycline	I
Trimethoprim/Sulfamethoxazole	S
